# Portal Vein Thrombosis Extending to the Superior Mesenteric and Splenic Veins Secondary to a Hypercoagulable State Due to Prior Abdominal Surgery, Hormonal Replacement Therapy, and Flaxseed Consumption

**DOI:** 10.7759/cureus.36252

**Published:** 2023-03-16

**Authors:** Carlos Gaibor, Zhaoqian Zhang, Mei Yang, Imran Haider

**Affiliations:** 1 Internal Medicine, St Luke's Hospital, Chesterfield, USA

**Keywords:** surgery, mesenteric venous thrombosis, thrombosis, testosterone, portal vein thrombosis

## Abstract

Herein is a case of a 50-year-old male diagnosed with a non-cirrhotic acute portal vein thrombosis (PVT). Acute PVT is a rare condition usually presenting in cirrhotic patients. This patient had no past medical history of cirrhosis or hypercoagulable status and no past family history of a hypercoagulable disorder. However, the patient, who has been on testosterone replacement therapy (TRT) along with over-the-counter flax seeds (commonly known to contain phytoestrogens), recently underwent an abdominal surgery essentially placing him in a hypercoagulable state which could contribute to the development of acute PVT. This case showed the importance of being aware of possible contributors to hypercoagulable states which can lead to the occurrence of these events.

## Introduction

Portal vein thrombosis (PVT) commonly occurs in patients with cirrhosis and/or prothrombotic disorders [1-3]. PVT in a patient without cirrhosis is a rare disease (an autopsy study from Japan showed a prevalence of 0.05%; however, this study likely overestimated PVT prevalence in the general population because of postmortem thrombosis) [2]. Acute PVT is caused by a thrombus occluding the portal vein partially or entirely with extension to the mesenteric and/or splenic vein. On the other hand, chronic PVT is suspected when the patient develops collateral circulation (e.g., cavernous portal transformation) or portal hypertension [4]. Moreover, when there is no information on the chronicity of the clot, the PVT can be referred to as recent and could be managed as acute PVT [1]. Prompt diagnosis and management are essential to prevent dreadful consequences like mesenteric ischemia, chronic cavernous transformation, and complications of portal hypertension. Its clinical presentation varies from asymptomatic, often diagnosed incidentally with an imaging study done for another reason [1], to sudden or progressive abdominal pain associated with nausea and dyspeptic symptoms. Similarly, when the superior mesenteric vein is involved, colicky abdominal pain associated with diarrhea could be present, and septic PVT may be suspected when spiking fever is present. The physical examination could be inconspicuous or abdominal distention could be present. Laboratory testing may show elevated acute-phase reactants. Acute PVT is diagnosed with abdominal imaging with contrast-enhanced abdominal computerized tomography (CT), but a Doppler ultrasound could be performed if the suspicion is low [5]. Acute PVT is managed with anticoagulation [6], such as low molecular weight heparin, to achieve rapid anticoagulation that can be switched to an oral anticoagulant once the patient's condition stabilizes. 

## Case presentation

The patient is a 58-year-old male who presented to the emergency department (ED) with epigastric pain that gradually started two days prior to presentation. The patient reported a significant past medical history of hypertension managed with lisinopril, hyperlipidemia managed with atorvastatin, sleep apnea, and a 10-year history of chronic anemia of unknown cause managed with ferrous sulfate oral tablets. His family history was not relevant for any bleeding diathesis or hypercoagulable disorders. In addition, the patient underwent a partial fundoplication due to a hiatal hernia with Cameron’s erosions a month prior to presentation. A proton pump inhibitor (PPI) was prescribed afterward, but it was discontinued upon symptom improvement. Additionally, the patient reported being on hormone replacement therapy (HRT) with low-dose testosterone for over a decade and was consuming flax seeds (commonly known to contain phytoestrogens) and omega-3 polyunsaturated fatty acid. The pain was intermittent, non-radiating, and fluctuating in intensity, was not exacerbated or relieved by any means, including PPI, and was associated with mild nausea and subjective fever. Otherwise, he denied chest pain, palpitations, shortness of breath, vomitus, diarrhea, constipation, and rectal bleeding. He received his fourth dose of the COVID vaccine two weeks prior to presentation. Physical examination was only conspicuous for a small umbilical hernia, but the abdomen was soft, non-distended with normal bowel sounds, and without organomegaly. Laboratory results (Table 1) were relevant for hemoglobin and INR levels of 18 gr/dl and 1.1, respectively. 

**Table 1:** Laboratory workup

Laboratory	Result	Normal range
Hemoglobin	18 gr/dl	13.6-16.5 gr/dl
INR (international normalized ratio)	1.1	0.9-1.1
PT (prothrombin time)	14.4 seconds	23.0-34.0 seconds
PTT (partial thromboplastin time)	29.2 seconds	11.9-14.9 seconds
BUN (blood urea nitrogen)	15 mg/dl	9-20 mg/dL
Creatinine	1.1 mg/dl	0.7-1.3 mg/dL
Albumin	4 gr/dl	3.5-5.0 gr/dl
Alkaline phosphatase	107 U/L	38-126 U/L
Total Bilirubin	1 mg/dl	0.2-1.3 mg/dl
AST (aspartate aminotransferase)	59 U/l	14-54 U/L
ALT (alanine transferase)	110 U/L	<= 50 U/L
Hemoglobin A1C	5.50%	4.0-6.0 %
Testosterone	56.4 ng/dl	72.0-623.0 ng/dl

**Figure 1:**
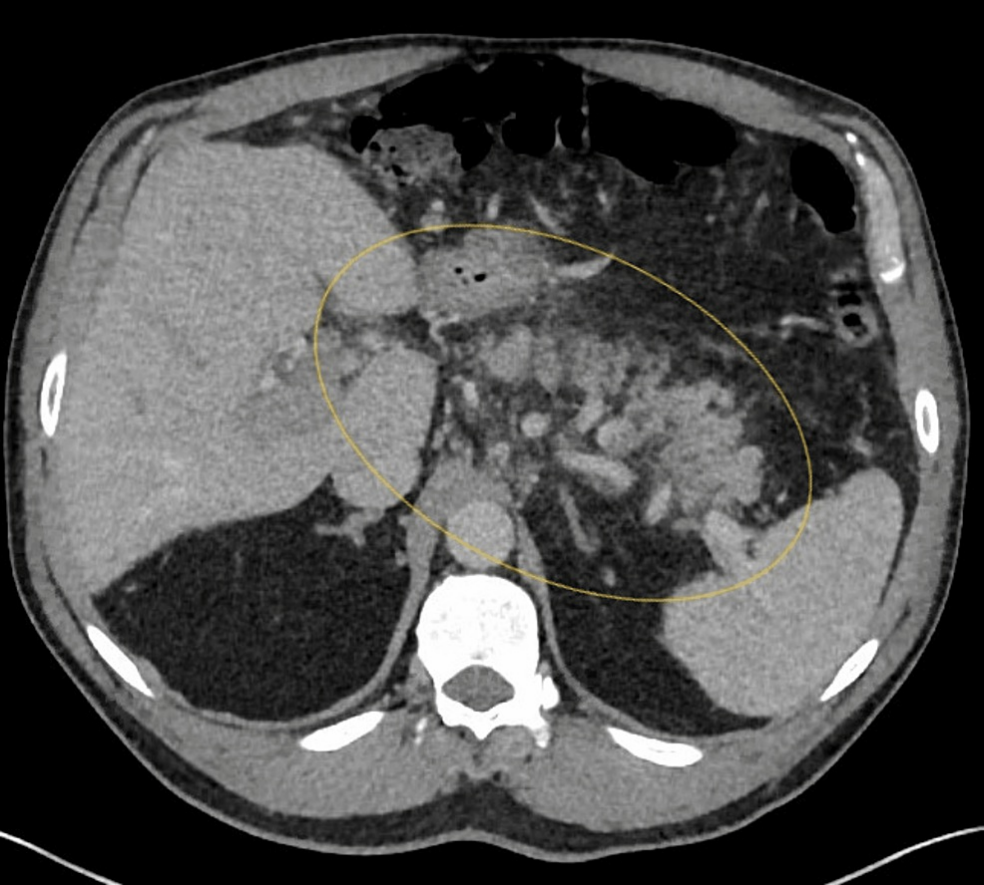
Computerized tomography of the abdomen and pelvis with contrast shows an extensive clot seen in the portal, splenic, and superior mesenteric veins (yellow circle).

**Figure 2:**
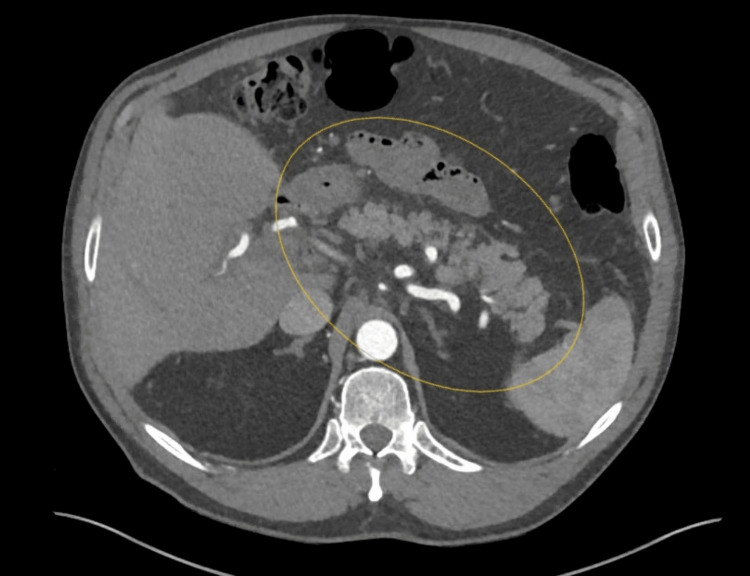
Computed tomography angiogram of the abdomen and pelvis confirms an extensive clot in the portal, splenic, and superior mesenteric veins (yellow circle).

Additionally, troponin was negative; an EKG was also negative for ischemic changes. Subsequently, computerized tomography with contrast of the abdomen and pelvis (Figure 1) demonstrated an extensive clot in the portal vein extending to the splenic and superior mesenteric veins, which was confirmed by a CT angiogram with contrast (Figure 2). As a result, a heparin drip was started, and hematology was consulted; they recommended commencing Eliquis, stopping HRT, and discharging the patient. After the discharge, a hypercoagulable workup was done and was negative for antithrombin III activity, factor II mutation, factor V Leiden mutation, protein C activity, protein S functional activity, and prothrombin mutation. Additionally, JAK2 was negative, and erythrocytosis normalized after testosterone was discontinued. 

## Discussion

This case report presents a rare case of a 50-year-old male who developed portal vein thrombosis (PVT) extending to the splenic and mesenteric veins. PVT is usually seen in cirrhotic patients; however, this patient did not have any history of liver disease. Furthermore, the patient did not report any family history of a hypercoagulable disorder like factor V Leiden; hypercoagulable workup was negative, including for JAK2, which has been reported as a contributor to PVT [7]. However, the patient has been on TRT for over a decade. Similarly, the patient mentioned consuming flaxseed (Linum usitatissimum) known for containing phytoestrogens called lignans that need to be further studied as a contributor to a hypercoagulable state. Therefore, it became evident, after ruling out any predisposing factors such as a history of cirrhosis or hypercoagulable disorder, that TRT and flaxseed consumption had played a role in increasing the risk of thrombosis in this patient. Consequently, his recent abdominal surgery was a trigger for the development of this condition. Taking everything into account, it could be concluded that TRT usage could be a risk factor for PVT in the setting of an abdominal insult such as, in this case, abdominal surgery. For this reason, this case report could help clinicians be cognizant of potential pro-thrombotic risk factors that normally are overlooked and to assist in the decision of initiating or extending anti-thrombotic prophylaxis in order to prevent dreadful consequences such as in this case. 

## Conclusions

This case showed the importance of being cognizant of potential hypercoagulable risk factors such as being postsurgical, long-term testosterone replacement usage, and consumption of over-the-counter supplements that could contribute to the development of acute PVT. It also raises the question regarding the role of prophylactic anticoagulation in reducing the risk of PVT. Therefore, it is important that TRT could be identified as a potential pro-thrombotic risk factor in order to prevent dreadful outcomes.
